# Functional near-infrared spectroscopy of medical students answering various item types

**DOI:** 10.3389/fpsyg.2023.1178753

**Published:** 2023-06-12

**Authors:** Syeda Fabeha Husain, Nixi Wang, Roger S. McIntyre, Bach X. Tran, Thao Phuong Nguyen, Linh Gia Vu, Giang Thu Vu, Roger C. Ho, Cyrus S. Ho

**Affiliations:** ^1^Institute of Health Innovation and Technology (iHealthtech), National University of Singapore, Singapore, Singapore; ^2^Department of Paediatrics, Yong Loo Lin School of Medicine, National University of Singapore, Singapore, Singapore; ^3^Mood Disorders Psychopharmacology Unit, University Health Network, Toronto, ON, Canada; ^4^Department of Psychiatry, University of Toronto, Toronto, ON, Canada; ^5^Department of Pharmacology, University of Toronto, Toronto, ON, Canada; ^6^Brain and Cognition Discovery Foundation, Toronto, ON, Canada; ^7^Bloomberg School of Public Health, Johns Hopkins University, Baltimore, MD, United States; ^8^Institute for Preventive Medicine and Public Health, Hanoi Medical University, Hanoi, Vietnam; ^9^Institute for Global Health Innovations, Duy Tan University, Da Nang, Vietnam; ^10^Faculty of Medicine, Duy Tan University, Da Nang, Vietnam; ^11^Institute for Global Health Innovations, Duy Tan University, Da Nang, Vietnam; ^12^Center of Excellence in Behavioral Medicine, Nguyen Tat Thanh University, Ho Chi Minh City, Vietnam; ^13^Institute of Health Economics and Technology, Hanoi, Vietnam; ^14^Department of Psychological Medicine, Yong Loo Lin School of Medicine, National University of Singapore, Singapore, Singapore

**Keywords:** case scenarios, multiple choice questions, short answer questions, true/false questions, functional near-infrared spectroscopy, item type, medical examination

## Abstract

**Background:**

Traditionally, the effect of assessment item types including true/false questions (TFQs), multiple-choice questions (MCQs), short answer questions (SAQs), and case scenario questions (CSQs) is examined through psychometric qualities or student interviews. However, brain activity while answering such questions or items remains unknown. Functional near-infrared spectroscopy (fNIRS) can be used to safely measure cerebral cortex hemodynamic response during various tasks. Hence, this fNIRS study aimed to determine differences in frontotemporal cortex activity as medical students answered TFQs, MCQs, SAQs, and CSQs.

**Methods:**

In total, 24 medical students (13 males and 11 females) were recruited in this study during their mid-psychiatry posting. Oxy-hemoglobin and deoxy-hemoglobin levels in the frontal and temporal regions were measured with a 52-channel fNIRS system. Participants answered 9–18 trials under each of the four types of tasks that were based on their psychiatry curriculum during fNIRS measurements. The area under the oxy-hemoglobin curve (AUC) for each participant and each item type was derived. Repeated measures ANOVA with post-hoc Bonferroni-corrected pairwise comparisons were used to determine differences in oxy-hemoglobin AUC between TFQs, MCQs, SAQs, and CSQs.

**Results:**

Oxy-hemoglobin AUC was highest during the CSQs, followed by SAQs, MCQs, and TFQs in both the frontal and temporal regions. Statistically significant differences between different types of items were observed in oxy-hemoglobin AUC of the frontal region (*p* ≤ 0.001). Oxy-hemoglobin AUC in the frontal region was significantly higher during the CSQs than TFQ (*p* = 0.005) and during the SAQ than TFQ (*p* = 0.025). Although the percentage of correct responses was significantly lower in MCQ than in the other item types, there was no correlation between the percentage of correct response and oxy-hemoglobin AUC in both regions for all four item types (*p* > 0.05).

**Conclusion:**

CSQs and SAQs elicited greater hemodynamic response than MCQs and TFQs in the prefrontal cortex of medical students. This suggests that more cognitive skills may be required to answer CSQs and SAQs.

## 1. Introduction

The theory examination is used to determine if a medical student has reached an acceptable level of clinical competence based on the objectives of training in a medical discipline. Common formats for theory examination items include true/false questions (TFQs), multiple-choice questions (MCQs), short answer questions (SAQs), and case scenario questions (CSQs). TFQs are binary items with true or false statements to assess a medical student’s knowledge (Pinckard et al., 2012). MCQs consist of an item stem and multiple options. MCQs are used to assess a medical student’s knowledge and ability to apply this knowledge over a wide range of content areas (Unit EEaA, 2019a). Some educators believed that SAQs are more challenging than MCQs (Pinckard et al., 2012). SAQs are items that can be answered in short phrases or a few short words (Unit EEaA, 2019b). Typically, SAQs contain instructions such as ‘list’ or ‘name’ suggesting that short responses are required, or “what” suggesting that short explanations are required. SAQs usually consist of a single item and space where the medical student can provide his or her answer for that question only. SAQs require recall of factual knowledge or indicate short responses. Some SAQs are designed with only one expected answer while some SAQs are designed with a list of possible answers. SAQs may have a lower level of complexity as compared to CSQs (Unit EEaA, 2019b). CSQs are more complex items and usually include several sub-questions that are linked to a given case scenario (Unit EEaA, 2019b). CSQs can elicit the application of knowledge or clinical reasoning to assess and manage a clinical scenario.

Previous research found that item formats or types significantly affected the difficulty level of items. For instance, open-ended items were easier and more discriminative than CSQs and MCQs (AlKhatib et al., 2020). (Farooqui et al. 2018) found a mild to moderate significant correlation between MCQs and SAQs for theory examinations for medical students. However, the evaluation of the usefulness of item types predominantly relies on psychometric properties. The utility of neuroimaging techniques in assessing item validity and medical education has been strictly limited. The application of neuroimaging methodology has gradually garnered interest in recent years to examine brain activation patterns across item types. (Durning et al., 2013) carried out a functional magnetic resonance neuroimaging (fMRI) study to evaluate brain hemodynamic response as physicians answered MCQs and thought about MCQs aloud. The authors reported higher focal activation in the motor cortex, bilateral prefrontal cortex, bilateral cerebellum, and basal ganglia as physicians thought about the MCQs aloud as opposed to answering MCQs (Durning et al., 2013). While neuroimaging in medical education research is scarce, further neuroimaging investigations can enhance our understanding of brain activation during assessment in response to different item types, thereby guiding assessment design and item development in medical education.

Functional near-infrared spectroscopy (fNIRS) is used to measure cerebral cortex activity non-invasively by detecting relative changes in oxy-hemoglobin and deoxy-hemoglobin (Lai et al., 2017). fNIRS signals have been recorded as participants did sport (Seidel-Marzi and Ragert, 2020) and surgical training (Khoe et al., 2020) while standing, and fist-edge-palm task (Kobayashi et al., 2021), verbal fluency test (Husain et al., 2021), Iowa gambling task (Husain et al., 2020), olfactory stimulation test (Ho et al., 2021) and viewing greenery images (Juanhong et al., 2017) while sitting. Unlike conventional neuroimaging techniques such as fMRI, fNIRS is economical, portable, less sensitive to motion artifacts and recordings can be conducted in natural body positions. These practical advantages allow neurocognitive studies to be carried out in ecologically valid environments with a natural response type (Soltanlou et al., 2018). Hence, fNIRS set-ups could simulate the actual theory examination condition adopted by most medical schools.

To our knowledge, the cerebral hemodynamic response as medical students answer the different item types in a theory examination has not been previously reported. The aim of this study was to compare frontotemporal cortex hemodynamic responses between TFQs, MCQs, SAQs, and CSQs that were based on the undergraduate psychiatry curriculum. We hypothesized that hemodynamic response at the prefrontal cortex would be higher during CSQs and SAQs compared to MCQs and TFQs.

## 2. Methods

### 2.1. Participants

In total, 24 medical students were included in this cross-sectional study. All the participants were fourth-year undergraduate medical students undergoing psychiatry rotation at the Department of Psychological Medicine, National University Hospital, the teaching hospital of the National University of Singapore. To ensure the participants were at the same stage of learning and free from examination stress, study visits were scheduled during the second and third week of the 6-week psychiatry posting. The participants had no previous history of psychiatric, neurological, or chronic medical diseases.

The study details were fully explained to the potential participants and their written informed consent was obtained. This study was performed according to the Declaration of Helsinki and the ethical principles in the Belmont Report. The medical students were informed that participation was voluntary. Agreement or refusal to participate did not have any effect on their posting grade. During the study visit, the participants completed a questionnaire on demographics and medical history, and their fNIRS measurements during the TFQ, MCQ, SAQ, and CSQ tasks were recorded. This study was approved by the National University of Singapore Institutional Review Board (protocol number NUS-IRB-2021-384).

### 2.2. Functional near-infrared spectroscopy measurements

A 52-channel fNIRS system (ETG-4000. Hitachi Medical Co., Tokyo, Japan) measured relative oxy-hemoglobin and deoxy-hemoglobin changes using two near-infrared light wavelengths (695 and 830 nm) (Yamashita et al., 1996). The emitter and detector optodes were arranged 3 cm apart. The area between each emitter and detector pair is called a channel. Anatomically, channels correspond to cortical regions 2–3 cm beneath the skin and scalp surface (Okada and Delpy, 2003). Optodes were placed on the forehead and scalp, with the lowest optodes placed along the T4-Fpz-T3 line, defined by the 10/20 system. This arrangement allowed for hemoglobin changes in the bilateral prefrontal cortex, frontopolar cortex, and the anterior regions of the superior and middle temporal cortex to be measured. These approximate channel locations are based on the anatomical craniocerebral correction of the international 10/20 system.

### 2.3. Tasks during fNIRS measurements

**Figure 1:**
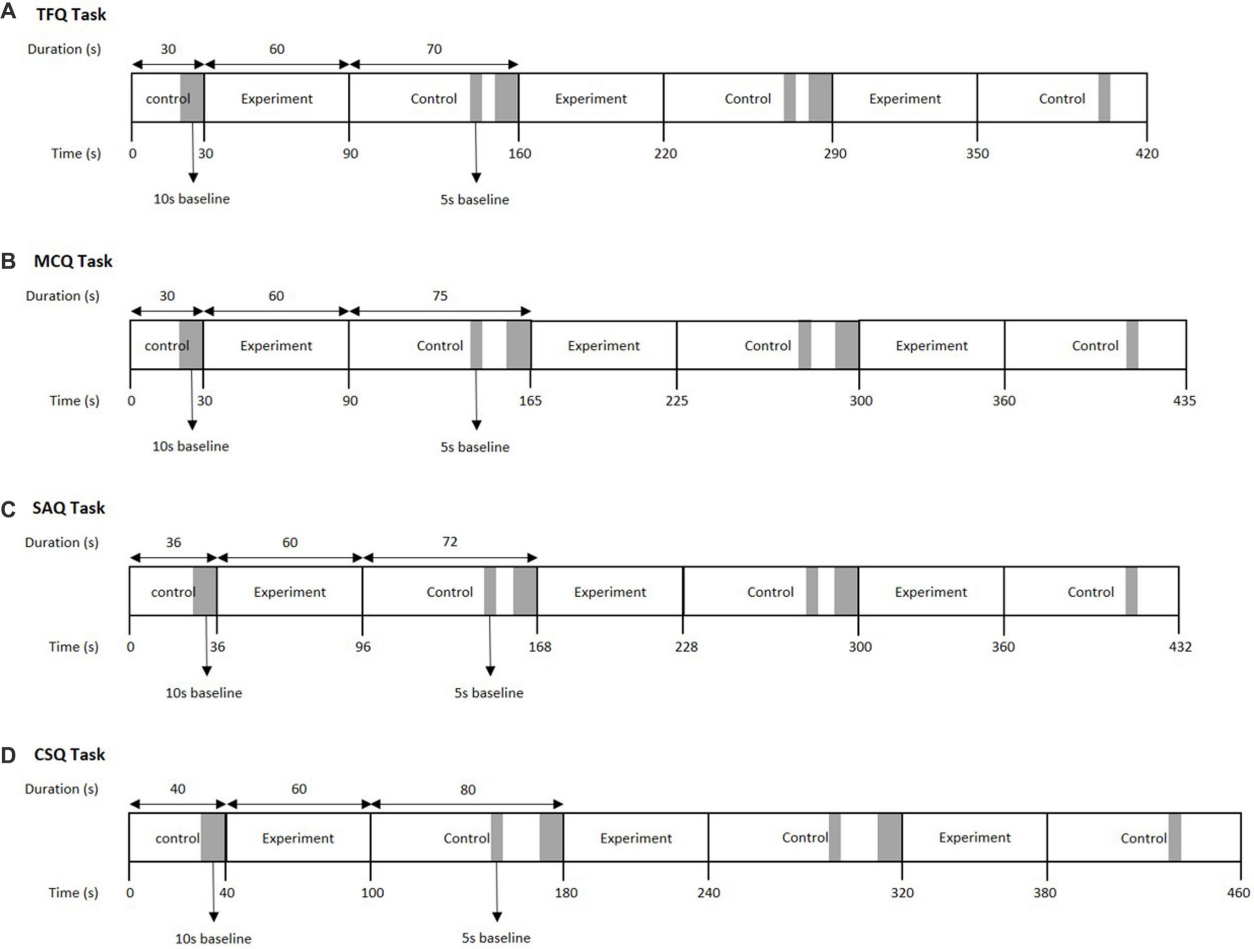
Task protocols for the **(A)** TFQ, **(B)** MCQ, **(C)** SAQ and **(D)** CSQ.

Functional near-infrared spectroscopy measurements were recorded as the participants performed tasks on E-Prime 3.0 (Psychology Software Tools Inc.). All the assessment items were based on the undergraduate psychiatry curriculum which covered psychopathology, schizophrenia, mood disorders, anxiety disorders, personality disorders, sleep disorders, psychosexual disorders, eating disorders, substance misuse disorders, child and adolescent psychiatry, old age psychiatry, forensic psychiatry, neuroscience, psychotherapy, and psychopharmacology. The participants did not attempt items used in this study prior to enrolment. The TFQ, MCQ, SAQ, and CSQ tasks consisted of four control blocks that alternated with three experiment blocks (Figure 1). The order of tasks was randomized. The experiment blocks were 60 s long for all tasks. The duration of the control blocks varied between tasks because the duration of a single trial differed between tasks.

The trials in the TFQ task were 10s long and started with a 9.5 s long stimulus followed by a 0.5 s long fixation cross display. During the control block trials, the participants clicked either true or false randomly. During experiment block trials, the participants were asked to judge whether a factual statement is true or false and respond by clicking true or false. The TFQ task consisted of 18 experiment trials in total. The following is an example of a TFQ displayed during an experiment trial:

*Conduct disorder is a common comorbidity of children diagnosed with attention deficit hyperactivity disorder (ADHD)*.

*True or False*.

*The correct answer is true*.

Trials in the MCQ task were 15 s long and started with a 14.5 s long stimulus followed by a 0.5 s long fixation cross display. During the control block trials, the participants randomly clicked the letter A, B, C, D, or E. During the experiment block trials, an MCQ item containing a stem that identifies or prompts a problem and possible responses were displayed. The response choices contained one correct answer and four distractors. The participants were instructed to click one of these responses. The MCQ task consisted of 12 experiment trials in total. The following is an example of an MCQ displayed during an experiment trial:

*Which of the following neuroanatomical areas is MOST implicated in the pathology of obsessive–compulsive disorder?*

- *Amygdala.*
- *Basal ganglia.*
- *Nucleus accumbens.*
- *Hippocampus.*
- *Substantia nigra.*

*The correct answer is B*.

Trials in the SAQ task were 12 s long and started with an 11.5 s long stimulus followed by a 0.5 s long fixation cross display. The participants were asked to type randomly during the control block trials and type their answers during the experiment block trials. The SAQ items displayed during experiment block trials were written in clear, simple, and direct language, and were specific about how the question should be answered. The answer was expected to be very short and the questions were very similar to very short answer items proposed by other educators and researchers (Sam et al., 2016; Scheeres et al., 2022). The SAQ task consisted of 15 experiment trials in total. The following is an example of an SAQ displayed during an experiment trial:

*Name ONE (1) medication to treat alcohol withdrawal?*

*Model answer: Diazepam*.

*Other acceptable answer: Chlordiazepoxide, Lorazepam for patients with liver impairment*.

Trials during the CSQ task were 20 s long and started with a 19.5 s long stimulus followed by a 0.5 s long fixation cross display. The participants were asked to type randomly during the control block trials and type their answers during the experiment block trials. One case scenario was used per experiment block and there were nine experiment trials in total. The following is an example of a CSQ displayed during an experiment trial:

*An 18-year-old woman came to see you for psychiatric problem. You notice that her body mass index (BMI) is 13.*

1. *What is the most important psychiatric diagnosis that you must consider?*
*Answer: Anorexia Nervosa.*
2. *State ONE (1) clinical feature that you must look for to support your diagnosis*.
*Answer: Any one of the following:*
  *Amenorrhea*.
  *Body imaging disturbance*.
  *Restrictive eating habit*.
  *Excessive exercise*.

The participant got one point for a correct answer and zero points for an incorrect answer. The proportion of correct responses (%) during each task was determined for each participant as a measure of task performance. As more than one correct answer was allowed in some SAQs and CSQs, these items were marked by a professor in psychiatry who was blinded to the identity of participants. He referred to the model answers set by the psychiatrists who wrote the SAQs and CSQs. Prior to fNIRS measurements, the participants completed practice trials to familiarise themselves with each task.

### 2.4. Functional near-infrared spectroscopy signal analysis

The modified Beer–Lambert law was used to derive changes in oxy-hemoglobin, deoxy-hemoglobin, and total hemoglobin from optical densities. Hemoglobin changes during the experiment blocks were normalized by linear fitting between 10s baselines before these blocks and 5 s baselines that began 50s after these blocks. A moving average factor of five was applied to remove short-term motion artifacts. An algorithm identifying channels with body movement artifacts, or high- and low-frequency noise was applied. Artifact channels were not used in further analysis (Takizawa et al., 2014).

**Figure 2:**
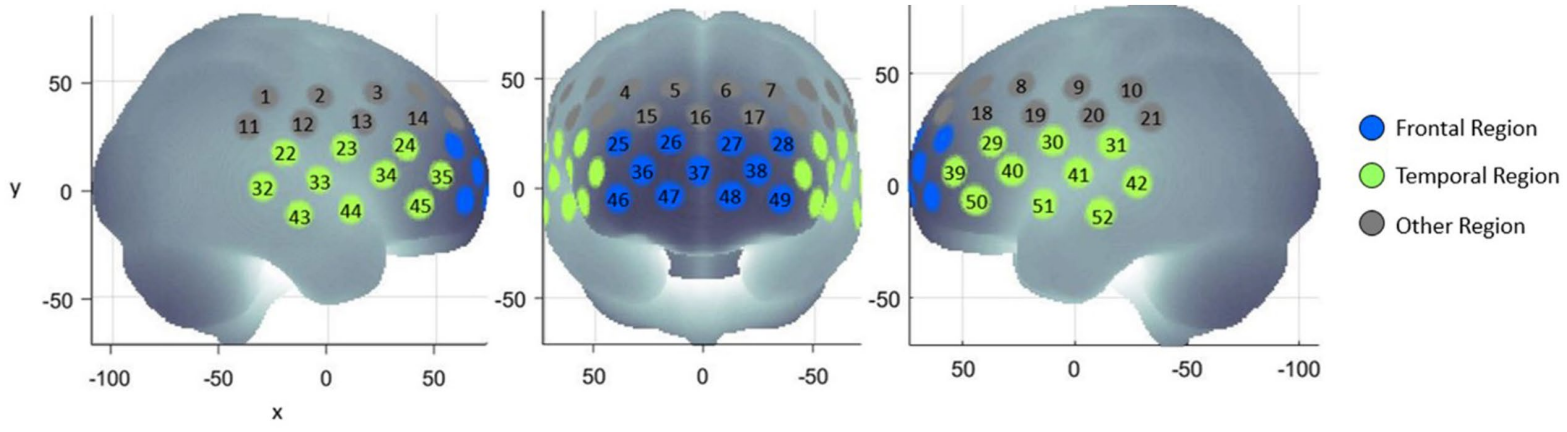
Frontal and temporal regions of interest.

Average oxy-hemoglobin and deoxy-hemoglobin waveforms of the three experiment blocks were generated for each region of interest and participant. The first region of interest comprised 11 channels located approximately at the frontopolar cortex and dorsolateral prefrontal cortex. The second region of interest comprised 20 channels located approximately at the left and right ventrolateral prefrontal cortex, superior temporal cortex, and middle temporal cortex. These regions were referred to as the ‘frontal region’ and ‘temporal region’, respectively (Figure 2). All the participants had at least six available channels in both regions of interest for each task. The hemodynamic response magnitude for each task, region, and participant was determined by calculating the oxy-hemoglobin area under the curve (AUC) during the experiment blocks (Takizawa et al., 2014).

### 2.5. Statistical analysis

Data assumptions were checked and a repeated measures ANOVA was conducted to examine the item type effect. Normal distribution and sphericity were assumed. In addition, post-hoc Bonferroni-corrected pairwise comparisons were conducted to determine differences in the oxy-hemoglobin AUC at each region and correct response rates between the four tasks. Effect size measures were reported along with the estimated marginal means for group differences. Hedges’ g statistic was used for its consideration of sample size. Pearson’s correlation was used to explore the relationship between the correct response rate and the oxy-hemoglobin AUC in each region for each task. All tests were two-tailed, with a significance level of *p* ≤ 0.05. Data are expressed as mean and standard deviation. Statistical analysis was done on SPSS Statistic 28 (IBM).

## 3. Results

**Figure 3:**
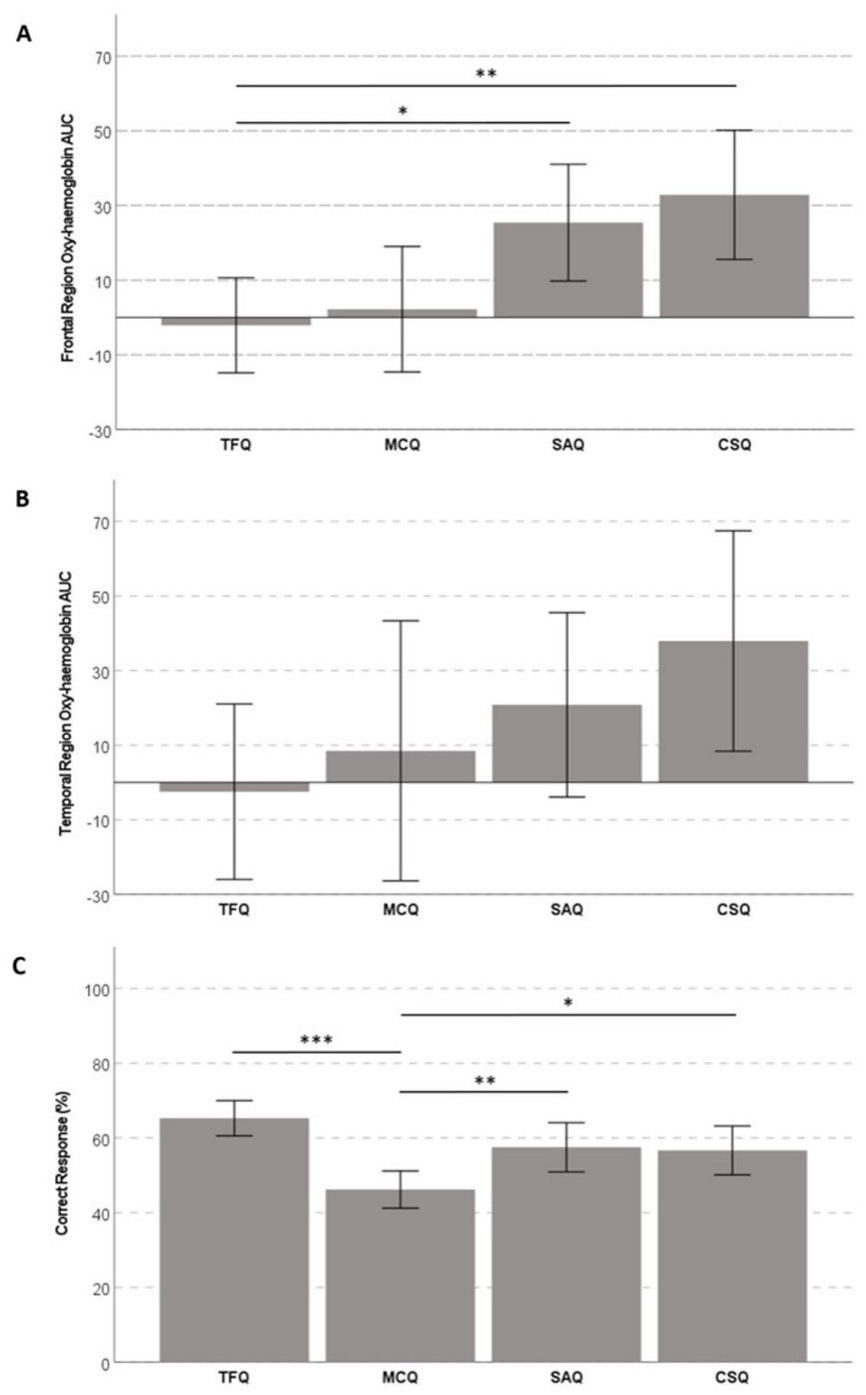
Comparison of **(A)** oxy-hemoglobin AUC in the frontal region, **(B)** oxy-hemoglobin AUC in the temporal region, and **(C)** percentage correct response between tasks (*p ≤ 0.05, **p ≤ 0.01, ***p ≤ 0.001).

**Figure 4:**
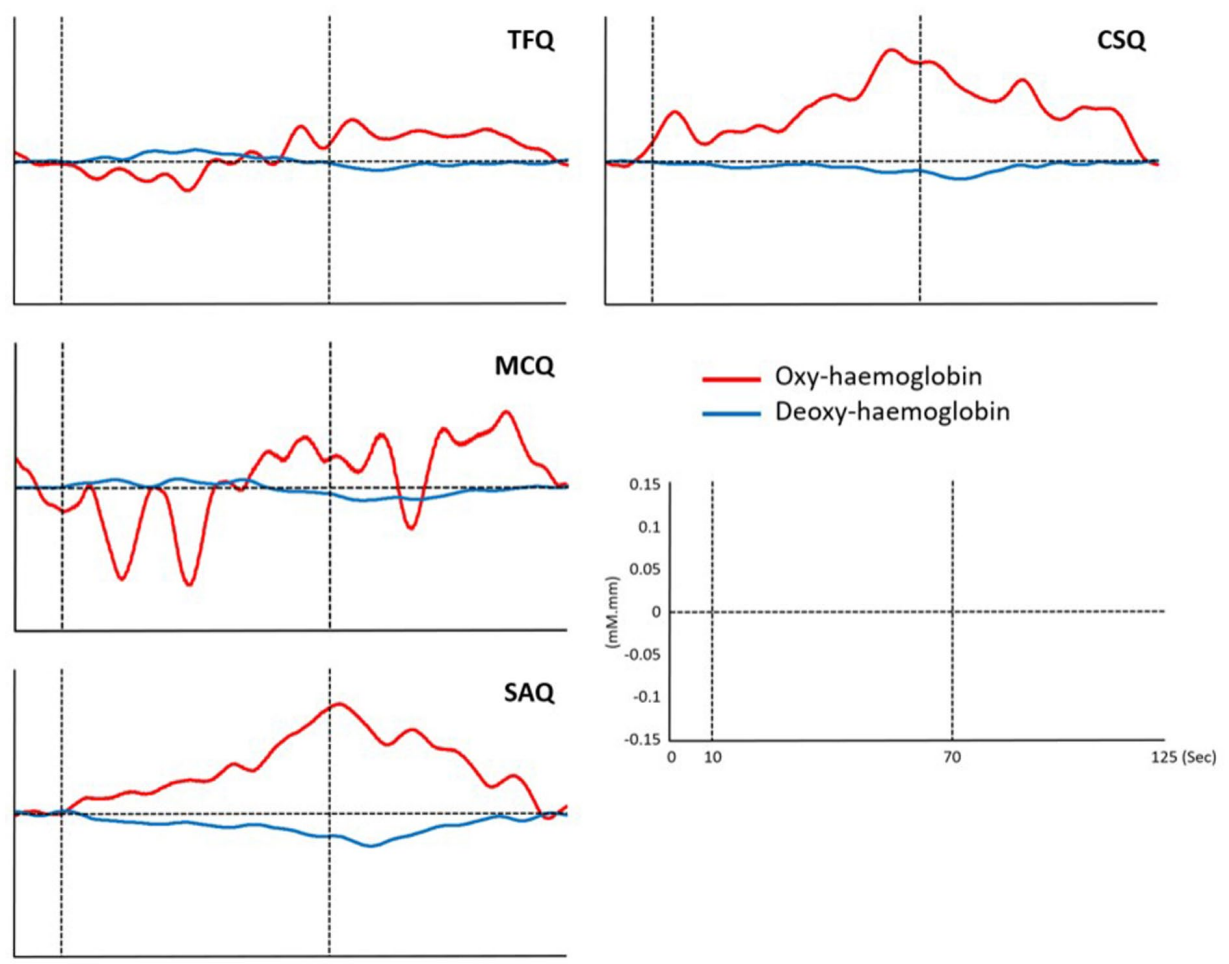
Average oxy-hemoglobin and deoxy-hemoglobin waveforms of the frontal region for each task. Vertical dotted lines indicate the start and end of the experiment block.

The oxy-hemoglobin AUC in the frontal region was highest during the CSQs, followed by the SAQs, MCQs, and TFQs (Table 1). Statistically significant differences were observed in the frontal region oxy-hemoglobin AUC between tasks with a large effect size [*F*(3, 69) = 7.21, *p* ≤ 0.001, partial $\eta^{2}$ = 0.24]. Specifically, the frontal region oxy-hemoglobin AUC during the CSQs was significantly higher than the TFQs [Mean difference = 34.95 (95% CI, 8.93–60.98), *p* = 0.005, Hedge’s *g* = 0.77], while the oxy-hemoglobin AUC in the frontal region during the SAQs was significantly higher than the TFQs [Mean difference = 27.50 (95% CI, 2.58–52.42), *p* = 0.025, Hedge’s *g* = 0.63] (Figures 3, 4).

**Table 1:** Descriptives of the current study sample.

	*N* (%)
Gender	
Male	13 (54.2%)
Female	11 (45.8%)
Ethnicity	
Chinese	23 (95.8%)
Mixed (Chinese and Indian)	1 (4.2%)
Handedness	
Right	23 (95.8%)
Left	1 (4.2%)
	Mean (SD)
Age	22.1 (0.9)
Frontal region oxy-hemoglobin AUC	
TFQ	−2.1 (30.2)
MCQ	2.2 (39.8)
SAQ	25.4 (37)
CSQ	32.9 (41)
Temporal region oxy-hemoglobin AUC	
TFQ	−2.5 (55.7)
MCQ	8.5 (82.5)
SAQ	20.8 (58.6)
CSQ	37.9 (70)
Correct response (%)	
TFQ	65.3 (11.1)
MCQ	46.2 (11.8)
SAQ	57.5 (15.6)
CSQ	56.7 (15.5)

Similar to the frontal region, the oxy-hemoglobin AUC in the temporal region was the highest during the CSQs, followed by the SAQs, MCQs, and TFQs. However, there were no statistically significant differences in the temporal region oxy-hemoglobin AUC between tasks [*F*(3, 69) = 2.28, *p* = 0.087] (Figures 3, 4).

The percentage of correct responses was the highest for the TFQs, indicating the easiest difficulty for this item type, followed by the SAQs, CSQs, and MCQs (Table 1). Moreover, statistically significant differences were found among the four types in task performance [*F*(3, 69) = 10.17, *p* ≤ 0.001, partial $\eta^{2}$= 0.04]. Specifically, the mean percentage of correct responses was higher for the TFQs than the MCQs [Mean difference = 19.10 (95% CI, 10.41 to 27.79), *p* ≤ 0.001, Hedge’s *g* = 1.25], higher for the SAQs than the MCQs [Mean difference = 11.32 (95% CI, 2.43 to 20.21), *p* = 0.008, Hedge’s *g* = 0.73] and higher for the CSQs than the MCQs [Mean difference = 10.49 (95% CI, 0.55 to 20.42), *p* = 0.034, Hedge’s *g* = 0.60] (Figure 3). However, the percentage of correct responses did not correlate with the oxy-hemoglobin AUC in the frontal and temporal regions for all the tasks (Table 2).

**Table 2:** Correlations among oxy-hemoglobin AUC and percentage of correct response (%).

	Frontal region		Temporal region	
	Pearson’s *r*	Value of *p*	Pearson’s *r*	Value of *p*
TFQ	−0.035	0.872	0.309	0.142
MCQ	0.216	0.310	0.355	0.089
SAQ	−0.131	0.542	0.187	0.381
CSQ	−0.014	0.950	0.101	0.639

TFQ, true/false question; MCQ, multiple choice question; SAQ, short answer question; CSQ, case scenario question.

## 4. Discussion

To our knowledge, this is the first functional neuroimaging study that provides evidence of higher prefrontal cortex activity during the CSQs and SAQs than the TFQs. As task performance did not correlate with the oxy-hemoglobin AUC, prefrontal cortex activity may not be linked to test outcomes (i.e., the grade of the test or examination), and may instead reflect the underlying cognitive processes that occur when answering items. Another notable observation was that the order of tasks from highest to lowest oxy-hemoglobin AUC in both frontal and temporal regions was (Pinckard et al., 2012) CSQs, (Unit EEaA, 2019a) SAQs, (Unit EEaA, 2019b) MCQs, and (Farooqui et al., 2018) TFQs. This suggests that answering CSQs and SAQs relies on greater frontal lobe function, which includes attention and concentration, judgment, planning, problem-solving, working memory, and greater temporal lobe function, which includes retrieval from memory and language comprehension (Puri, 2013).

The observation that the highest frontotemporal hemodynamic response occurred during the CSQ task further supports postulations by medical educators in the past 40 years, namely that the CFQ employs more cognitive skills than other item types. In 1972, CSQs were proposed to enhance the clinical reasoning of undergraduate medical students, postgraduate trainees, and physicians (Elstein et al., 1972). In the 1980s and 1990s, researchers proposed that undergraduate medical students or postgraduate trainees use problem-solving (Coderre et al., 2004), thinking aloud (Skakun et al., 1994), and diagnostic reasoning (Coderre et al., 2003) strategies when answering CSQs (Heist et al., 2014).

The second highest frontotemporal oxy-hemoglobin AUC was observed during the SAQ task, and this may be attributed to the open-ended nature of SAQs, which encourage clinical reasoning and knowledge with cognitive flexibility. Both CSQs and SAQs are ideal assessments for medical students as they encourage deep learning, compared to mere recognition of the correct answer in MCQs (Wood, 2009). These item types require medical students to generate knowledge in the absence of cues, an approach that is more representative of clinical practice and patient care (Sam et al., 2016). The construction of answers during SAQs and CSQs may therefore produce long-lasting memories that benefit their future clinical practice (Wood, 2009).

The lower frontotemporal hemodynamic response during the MCQs and TFQs may reflect the mechanical or habitual recall of knowledge that is assessed by these item types. MCQs assess recognition memory, and recall is often affected by the cueing effect of the item. Medical educators may find it difficult to create good and valid distractors, while poorly constructed distractors make guessing easier. As a result, medical students may find it easier to answer MCQs compared to SAQs and CSQs. Yet, MCQs are thought to be superior to TFQs because TFQs have a 50% chance of guessing the right answer (for MCQs, there is only a 20% chance) and offer little insight as to why a student answered correctly. Thus, TFQs are best suited to assessing surface-level medical knowledge (Scheeres et al., 2022). As frontotemporal activity during the MCQs was higher than the TFQs, this observation further supports the postulation that MCQs are more stimulating than TFQs.

Our findings have implications for future theory assessments and examinations for undergraduates, postgraduate residents, and trainees. For undergraduates, Sam et al. (2016) believed that reliance on assessment by single best answer MCQs (e.g., best out of 5 answers) could foster the learning of association and superficial understanding of medical knowledge in order to pass exams (Sam et al., 2016). For postgraduate trainees or residents, Scheeres et al. (2022) proposed the gradual introduction of SAQs to enable a more comprehensive assessment of their core medical knowledge and promote deeper learning strategies. A carefully phased introduction of alternative item formats, such as CSQs and SAQs, would enable a more robust assessment and provide opportunities for improving the assessment of theoretical knowledge (Scheeres et al., 2022). The development of artificial intelligence or machine learning algorithms that can mark SAQs and CSQs would reduce manpower needs. Examinations using SAQs and CSQs could then be cost-effective, especially in national and high stake postgraduate examinations that involve a large number of candidates (e.g., Postgraduate Royal College Exams in the United Kingdom, Australia, Canada, and the Commonwealth).

This study has several limitations, starting with the small sample size. This may explain why differences in the temporal region oxy-hemoglobin AUC between different item types did not reach statistical significance. Additionally, this may not be a representative sample as the participants were recruited from a single teaching hospital. Secondly, items used only covered psychiatry to avoid other confounding factors including inter-disciplinary variations. A future assessment covering other medical or surgical disciplines is needed, which may be extended to include postgraduate trainees and residents. Thirdly, the study set-up may not have reflected actual examination conditions because participants knew that their performance during tasks had no impact on their actual posting scores. Nevertheless, starting with such an investigation across item conditions point to potential future applications and development in this field.

## 5. Conclusion

This preliminary functional neuroimaging study provides evidence that prefrontal cortex activity varies between different item types in medical education assessment. As the hemodynamic response was not associated with task performance, these results suggest that CSQs and SAQs may require more cognitive skills from the frontal lobe compared to MCQs and TFQs. These findings need to be replicated in other medical or surgical disciplines and in a larger sample of medical students or postgraduate trainees.

## Data availability statement

The raw data supporting the conclusions of this article will be made available by the authors, without undue reservation.

## Ethics statement

The studies involving human participants were reviewed and approved by National University of Singapore Institutional Review Board (protocol number NUS-IRB-2021-384). The patients/participants provided their written informed consent to participate in this study.

## Author contributions

RM, RH, and CH: conception. SH and BT: design. SH: acquisition. NW, TN, LV, and GV: analysis. RH: interpretation of data for the work. SH and RH: drafting the work. NW, RM, BT, TN, LV, GV, and CH: critical revision. All authors contributed to the article and approved the submitted version.

## Funding

This study was funded by NUS Department of Psychological Medicine (R-177-000-100-001/R-177-000-003-001), NUS iHeathtech Other Operating Expenses (R-722-000-004-731), Vingroup Innovation Foundation (VINIF) (VINIF.2019.DA14) and in-kind contribution from the Fujifilm Healthcare Asia Pacific. The funding bodies had no role in the design of the study and collection, analysis, and interpretation of data and in writing the manuscript.

## Conflict of interest

The authors declare that the research was conducted in the absence of any commercial or financial relationships that could be construed as a potential conflict of interest.

## Publisher’s note

All claims expressed in this article are solely those of the authors and do not necessarily represent those of their affiliated organizations, or those of the publisher, the editors and the reviewers. Any product that may be evaluated in this article, or claim that may be made by its manufacturer, is not guaranteed or endorsed by the publisher.
